# PLOD2 increases resistance of gastric cancer cells to 5-fluorouracil by upregulating BCRP and inhibiting apoptosis

**DOI:** 10.7150/jca.41828

**Published:** 2020-03-13

**Authors:** Xiaohui Wang, Jiaojiao Guo, Meng Dai, Tengqi Wang, Tingting Yang, Xuejun Xiao, Qi Tang, Lingli Zhang, Lizhou Jia

**Affiliations:** 1 Cancer Center, Bayannur Hospital, Bayannur, Inner Mongolia, PR China.; 2 Department of Pharmacy, Inner Mongolia Medical University, Hohhot, Inner Mongolia, PR China.; 3 Department of Pathology, Nanjing Medical University, Nanjing, Jiangsu Province, PR China.; 4 Bayannur Clinical Medical College, Inner Mongolia Medical University, Bayannur, Inner Mongolia, PR China.; 5 Department of Pharmacology, Xinjiang Medical University, Wulumuqi, Xinjiang, PR China.; 6 Department of Ophthalmology, Inner Mongolia Autonomous Region People's Hospital, Hohhot, PR China.

**Keywords:** Gastric cancer, 5-Fluorouracil, PLOD2, drug resistance, BCRP (ABCG2)

## Abstract

**Background:** Gastric cancer (GC) is one of the most common cancers, and it is the third most common cause of cancer-related mortality worldwide. Fluorouracil (5-FU)-based chemotherapy is frequently used for the treatment of advanced GC. However, a substantial proportion of patients eventually experience refractory disease due to drug resistance. PLOD2 was reported to increase invasion and migration in several GC cell lines, but the roles of PLOD2 in chemoresistance are still unclear. The present study aimed to determine whether PLOD2 could confer 5-FU resistance in GC.

**Methods:** The expression of PLOD2 in GC cell lines was assessed by Western blotting. The cells were transfected by lentiviral transduction. The IC50 values were determined by the CCK-8 assay. The migration and invasion abilities of cells were analyzed by the Transwell assay. The proportion of apoptotic cells was assessed by flow cytometry. The protein levels of P-gp (MDR1), MRP1, BCRP (ABCG2), Bax and Bcl2 were analyzed by Western blotting. Furthermore, tumor xenograft models in nude mice were established to test tumor growth and weight.

**Result:** The knockdown of PLOD2 in BGC823 cells significantly decreased the IC50 values of 5-FU. It also contributed to reducing the cell migration and invasion and promoting the apoptosis of GC cells. The opposite results appeared in PLOD2-overexpressing MGC803 GC cells. *In vivo* experiments showed that the knockdown of PLOD2 increased the growth inhibition of transplanted tumors in nude mice in response to 5-FU. Our mechanistic studies revealed that PLOD2-overexpressing cells appear to be resistant to the therapeutic characteristics of 5-FU in GC cells by upregulating BCRP and that PLOD2 confers resistance to 5-FU-induced apoptosis in GC cells by affecting the expression of Bax and Bcl2.

**Conclusion:** PLOD2 contributed to increasing resistance of gastric cancer cells to 5-fluorouracil by upregulating BCRP and inhibiting apoptosis.

## Introduction

Gastric cancer (GC) is reported as the fifth most commonly diagnosed cancer and is the third most common cause of cancer-associated death in the world 1. At present, the treatment of gastric cancer mainly includes surgical intervention and chemotherapy/radiotherapy treatment 2. Among many types of drugs are applied in GC treatment, 5-fluorouracil (5-FU)-based chemotherapy is the mainstream therapeutic strategy 3. Accumulating evidence of the molecular mechanism of 5-FU in cancer inhibition has contributed to the development of therapies 4. One way in which 5-FU exerts its antitumor effect is through abnormal RNA processing, and the other is through inhibition of DNA synthesis, which is considered to be the main mechanism of its antitumor effect 5. Although advances in chemotherapy have reduced the mortality rate of GC patients, GC remains a major global public health problem 6. One of the main reasons is that drug resistance still frequently occurs during chemotherapy, which is a key barrier in the efficacy of GC treatment 7. Therefore, finding new strategies for potentiation of the current therapeutic approaches or potential targets is an urgent clinical necessity.

Extracellular matrix (ECM) remodeling is a typical feature of the tumor microenvironment and plays a vital role in aspects of tumor progression, including differentiation, proliferation 8, migration, adhesion, and survival 9. Collagen is one of the major components of the ECM, providing the scaffold for ECM assembly 10. Increased collagen deposition and cross-linking promote ECM stiffening, resulting in enhanced cancer cell migration and invasion 11. Lysyl hydroxylase 2 (LH2, encoded by the PLOD2 gene) is the key enzyme mediating the formation of stabile collagen cross-links 12. Therefore, PLOD2 may contribute to cancer progression by modulating collagen cross-linking and maturation 13. As previously reported, compared with normal breast tissues, PLOD2 expression was significantly increased in breast cancer tissues and was associated with short disease-related survival 14. As a diagnostic factor for hepatocellular carcinoma (HCC), PLOD2 is positively correlated with disease-free survival time and significantly correlated with tumor size 15. PLOD2 is elevated in non-small-cell lung cancer (NSCLC) specimens compared to normal specimens and is positively correlated with poor prognosis of NSCLC. PLOD2 directly promotes NSCLC metastasis by enhancing migration and indirectly promotes NSCLC metastasis by inducing collagen recombination 16. Among the GC patients, the PLOD2 high group had large amounts of collagen in their cancer tissues. PLOD2 knockdown through siRNA in several GC cell lines suppresses invasion and migration *in vitro* 17.

Although PLOD2 expression is reportedly associated with poor prognosis in several types of cancer 14-17, its role in chemoresistance remains unknown. In the following study, we investigated the effect of PLOD2 on the sensitivity of GC cells to 5-FU. The relative expression levels of PLOD2 in GC cell lines were first evaluated; and relatively low-expressing MGC803 cells and relatively high-expressing BGC823 cells were selected for subsequent experiments. MGC803 cells stably transfected with Lentivirus-mediated PLOD2 had increased resistance to 5-FU, and BGC823 cells with PLOD2 knockdown by shRNA had decreased resistance to 5-FU. PLOD2 increased resistance to 5-FU by upregulating BCRP and inhibiting cell apoptosis induced by 5-FU through regulating the expression of Bax and Bcl2, leading to drug resistance. Taken together, these findings provide a theoretical and experimental basis for the future study of drug resistance in the clinical chemotherapy of GC.

## Materials and Methods

### Cell culture and lentivirus infection

Seven human GC cell lines (MKN45, MKN28, MGC803, SGC7901, HGC27, and BGC823) and a normal human gastric epithelial cell line (GES-1) were maintained in our laboratory. Cell lines were cultured in DMEM with 10% fetal bovine serum and 1% penicillin-streptomycin (Gibco, USA) at 37°C in a humidified atmosphere containing 5% CO2. Overexpression plasmid pLenti-CMV-PLOD2-Flag-GFP-Puro, its control plasmid pLenti-CMV-GFP-Puro, knockdown plasmid pPLK/GFP+Puro-PLOD2 and its control plasmid shRNApPLK-GFP-Puro were all synthesized from public protein/plasmid libraries. The overexpression plasmid target gene length is 2238 bp (Gene ID: 5352; Accession Number: NM_000935). The knockdown plasmid target gene length is 61 bp (Gene ID: 1563; Accession Number: NM_000905). The lentiviruses were produced by transfecting 293T cells with Lipofectamine 2000 (Invitrogen, USA) following the manufacturer's protocol. The positive cell population was selected using puromycin, and transfection efficiency was determined by FACS, RT-qPCR and Western blot analysis. Then, the cells were treated with or without 1 μM 5-FU for 48 hours in the following experiments.

### Real-Time Quantitative PCR (RT-qPCR)

Total RNA was extracted from GC cell lines by the Trizol method (Invitrogen, USA) and then reverse transcribed into cDNA using the HiScript II RT Reagent Kit (Vazyme, China) according to the manufacturer's instructions. RT-qPCR was performed on an Applied Biosystems (ABI) StepOne Plus Sequence Detection System (Applied Biosystems, USA) in 96‐well plates. The PLOD2 mRNA expression levels were normalized to those of GAPDH. The primers used were F: 5'-AATGGGCAGCCGTTAGGAAA-3' and R: 5'-GCGCCCAATACGACCAAATC-3' for GAPDH and F: 5'-TCCCAAAGCTAAGTGCAGGC-3' and R: 5'-CACGTCTGGACTGTTTGCTC-3' for PLOD2. The Ct value for each sample was calculated using the ΔΔCt method, and the results were expressed as 2-ΔΔCt.

### Western Blot analysis

Cultured cells were washed twice in PBS and lysed in RIPA buffer (Beyotime, China) with complete protease inhibitors (Roche, Germany). Protein samples were separated by SDS-PAGE and transferred to PVDF membranes. Membranes were blocked with 5% low-fat dry milk in TBST for 2 hours at room temperature and probed with the indicated primary antibodies overnight at 4°C; Antibodies for the following targets were used: PLOD2 (Proteintech, USA), Bax (Signalway Antibody, USA), P-gp(MDR1), MRP1, BCRP(ABCG2), Bcl2 (Santa Cruz, USA), and GAPDH antibody (Abcam, USA). After washed with TBST, the membranes were incubated with HRP-conjugated anti-IgG secondary antibody at room temperature for 1 hour. Signals were analyzed using an Enhanced Chemiluminescence (ECL) Detection System (Tanon, China).

### Migration and invasion assay

Cell migration was evaluated using Transwell inserts with a pore size of 8 μm (BD Biosciences, USA). To investigate cell invasion, Transwell inserts were coated with Matrigel (BD Biosciences, USA) for 2 hours at 37°C. In both assays, 5×10^5^ cells were plated in the top chamber in medium without serum; the lower chamber was filled with 10% FBS or 1 μM 5-FU (DongXu, China). After 48 hours, migrated cells were stained with crystal violet, photographed, and counted using ImageJ software.

### CCK-8 assay

Cells (5 × 10^3^/100 μL) were seeded in 96-well plates (Corning, USA). After 12 hours, cells were cultured with 5-FU at various concentrations (0, 0.25, 0.5, 1, 1.5, 2, 3, 4, 5μM) for 48 hours. After the indicated treatments, cell viability was analyzed by using the CCK-8 Kit (Dojindo Laboratories, Japan) according to the manufacturer's instructions. The absorbance at 450 nm was measured on an enzyme-labeling instrument (Thermo, USA). The inhibitory rates of each cell line with different treatments were calculated by comparing the OD values of the experimental groups with that of the blank group. IC50 values (drug concentration causing 50% inhibition of cell growth) were calculated by using GraphPad Prism 7 software (San Diego, USA).

### Flow cytometry

After trypsinization, the cells were washed twice with cold PBS and then suspended (10^6^ cells/400 µl) in 1X binding buffer prior to incubation with 5 µl of APC Annexin V and 5 µl of propidium iodide (PI) solutions, according to the manufacturer's protocol for the APC Annexin V Apoptosis Detection Kit (BD Biosciences, USA). The cells were gently vortexed and incubated for 15 min at room temperature in the dark. The cells were analyzed using a FACS Aria flow cytometer (BD Biosciences, USA).

### Tumor xenografts in nude mice

All animal experiments were performed in accordance with the Institutional Committee for Animal Research and national guidelines for the care and use of laboratory animals. Twenty-four six-week-old BALB/c nude male mice (purchased from WTLH Company, China) were subcutaneously injected with 10^7^ NC-BGC823 or shPLOD2-BGC823 cells. When the tumor volumes reached 100-150 mm^3^ after 10 days, nude mice were randomly subdivided into four groups with 6 mice in each group. Experimental groups were treated with 5-FU intraperitoneally (50 mg/kg) once every two days for two weeks, while control groups were treated with PBS. After 4 weeks, nude mice were sacrificed by cervical dislocation, and xenograft tumors were harvested and weighed. The xenograft tumor volumes were measured by caliper and calculated as V = 1/2 × (length×width^2^). Tumors were dissected and fixed in 10% formalin and embedded in paraffin to detect PLOD2 expression by immunochemistry.

### Immunohistochemistry

Paraffin sections were deparaffinized using dimethylbenzene, and the slides were hydrated with different concentrations of alcohol. After endogenous peroxidase activity was blocked using 3% hydrogen peroxide, antigen retrieval was done by microwaving slides in 10 μM citrate buffer for 2 min. The rabbit anti-human PLOD2 antibody was diluted 1:50 and incubated with the samples overnight at 4°C. Samples were then incubated with a biotin-labeled rabbit anti-goat horseradish peroxidase-conjugated secondary IgG antibody for 1 hour at room temperature. The sections were washed with PBS twice and visualized with diaminobenzidine (DAB) and then counterstained with hematoxylin for 3 min. Dimethylbenzene was used to clean and seal the sections, which were then analyzed using a microscope.

### Statistical analysis

All statistical analyses were carried out using SPSS 20.0 software. The differences between each group were estimated by Student's t test, and GraphPad Prism 7.0 was used to draw all the plots. The results are presented as the mean±SD. P<0.05 was determined to represent a significant difference.

## Results

### Expression levels of PLOD2 in GC cell lines

**Figure 1:**
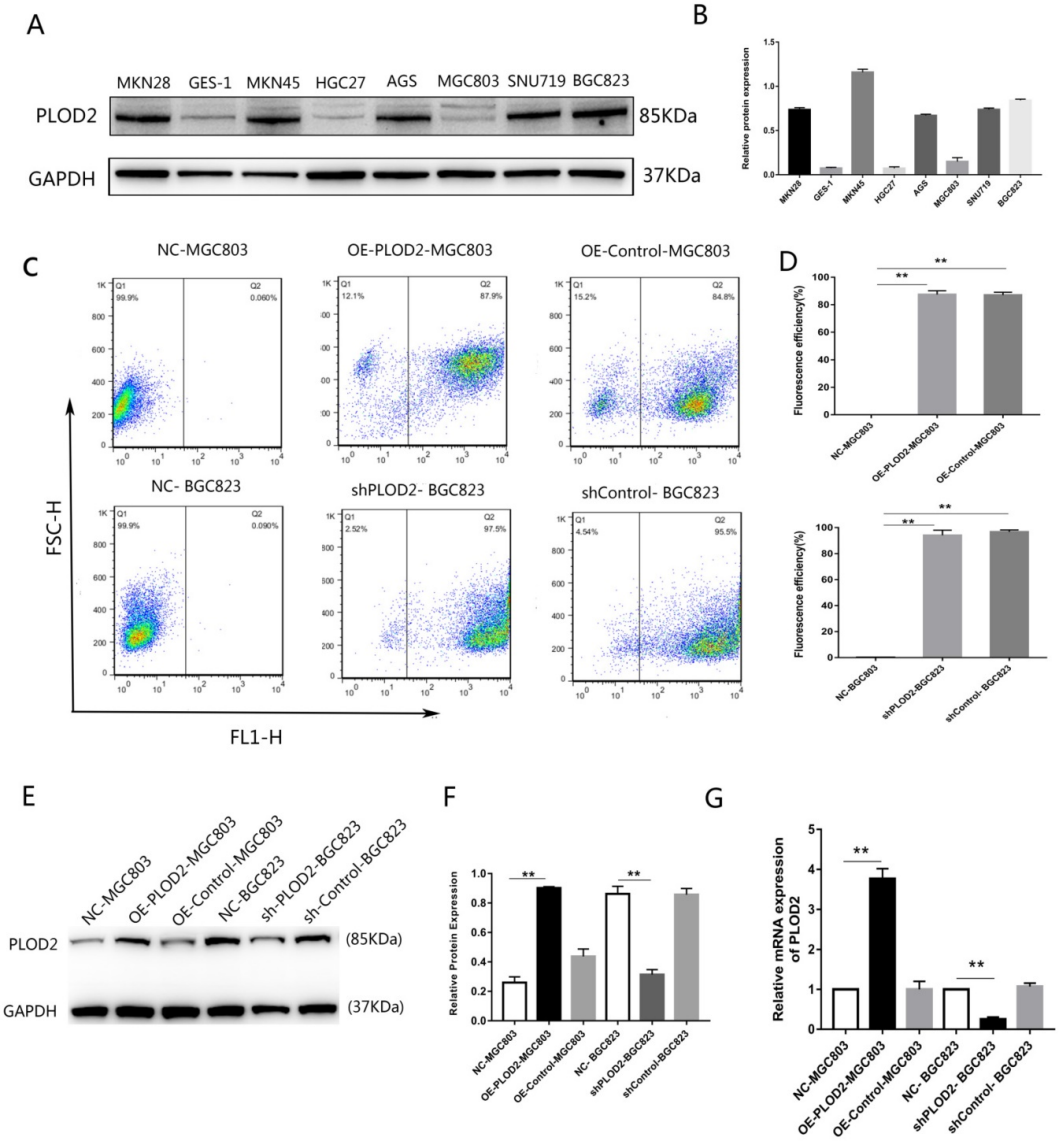
** Expression levels of PLOD2 in GC cell lines. (A,B)** The protein levels of PLOD2 in GC cell lines (MKN45, MKN28, MGC803, SGC7901, HGC27 and BGC823) and a normal human gastric epithelial cell line (GES-1) were analyzed by Western blot. **(C,D)** The fluorescence transfection efficiency of MGC803 and BGC823 cells after transfected with lentivirus infection determined by FACS. **(E,F)** The protein levels of PLOD2 in MGC803 and BGC823 cells transfected with lentivirus infection were analyzed by Western blot. **(G)** The mRNA levels of PLOD2 in MGC803 and BGC823 cells after transfected were analyzed by RT-qPCR. The results represent the mean ± SD of 3 independent experiments. *P < 0.05, **p < 0.01.

First, the relative expression level of PLOD2 was assessed in the normal gastric epithelial cell line GES-1 and GC cell lines. The expression of PLOD2 was significantly higher in BGC823, MKN45, MKN28, AGS and SNU719 cells than in MGC803, HGC27 and GES-1 cells (Figure 1A,B). The relatively low-expressing MGC803 cells and the relatively high-expressing BGC823 cells were used in the following experiment. To elucidate the potential roles of PLOD2 in chemoresistance, we successfully overexpressed PLOD2 in MGC803 cells and knocked down PLOD2 expression in BGC823 cells. The efficiency of transfection was significant as shown in (Figure 1C, D). RT-qPCR and Western blot analyses revealed that PLOD2 expression dramatically increased at both the mRNA and protein levels in OE-PLOD2-MGC803 cells compared to NC and OE-Control MGC803 cells but conversely decreased in shPLOD2-BGC823 cells compared to NC and shControl BGC823 cells (Figure 1E, F and G).

### PLOD2 enhances chemoresistance to 5-FU in GC cells by upregulating BCRP

**Figure 2:**
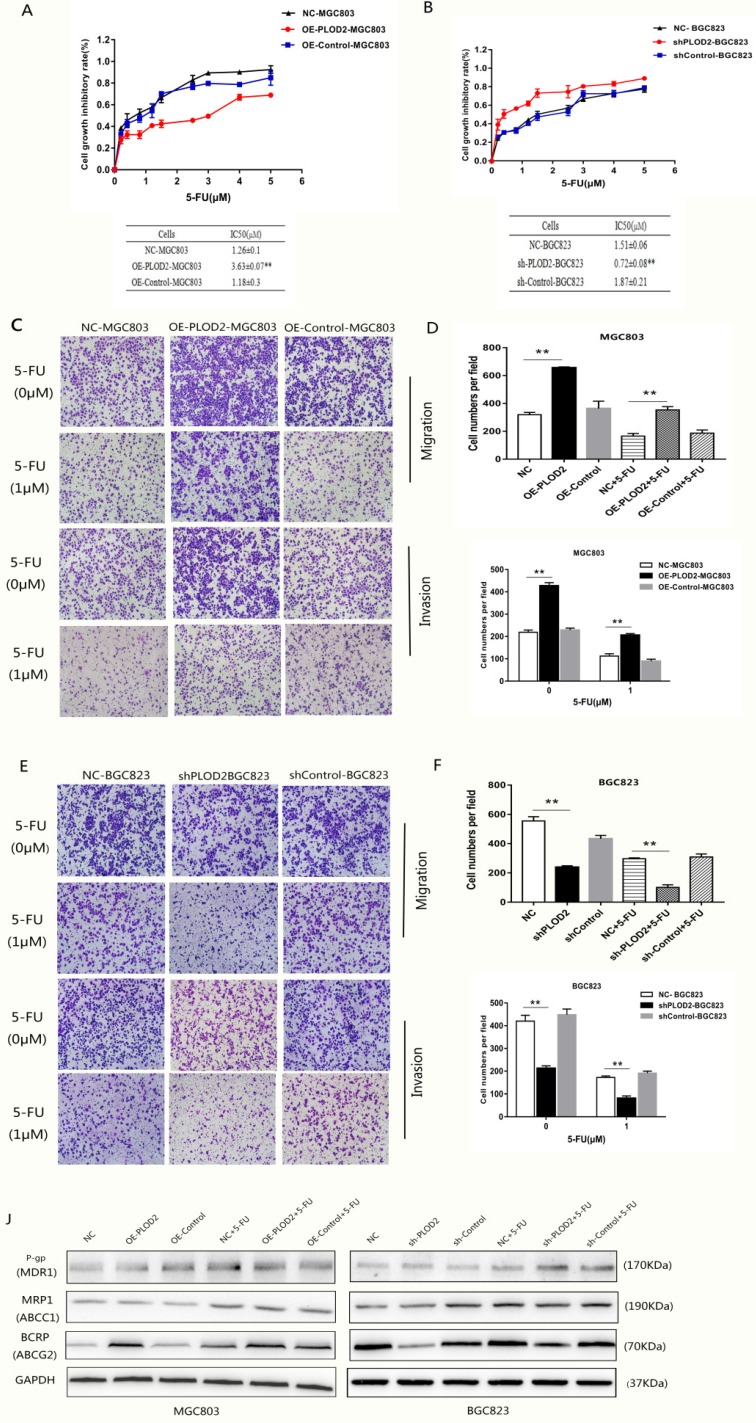
** PLOD2 enhances chemoresistance to 5-FU in GC cells by upregulating BCRP (A)** Upper: cell proliferation inhibitory curves of MGC803 cells upon 5-FU treatment. Lower: IC50 values of MGC803 cells upon 5-FU treatment. **(B)** Upper: cell proliferation inhibitory curves of BGC-823 cells upon 5-FU treatment. Lower: IC50 values of BGC-823 cells upon 5-FU treatment. **(C)** Transwell assays assessed the changes in migration and invasion of MGC803 cells that were treated with or without 5-FU. **(D)** Three fields for each chamber were photographed and the cells were counted. **(E)** Transwell assays assessed the changes in migration and invasion of BGC823 cells that were treated with or without 5-FU. **(F)** Three fields for each chamber were photographed and the cells were counted. **(J)** Western blot analysis of P-gp, MRP1 and BCRP(ABCG2) in MGC803 and BGC823 cells with or without 5-FU treatment. The results represent the mean ± SD of 3 independent experiments. *P < 0.05, **p < 0.01.

To determine whether PLOD2 confers chemoresistance in GC cells, the cells were treated with different concentrations of 5-FU, and the cell viability was analyzed. The results showed that PLOD2 overexpression decreased the sensitivity of MGC803 cells to 5-FU, as demonstrated by the increase in IC50 in OE-PLOD2-MGC803 cells compared to that in NC and OE-Control MGC803 cells (Figure 2A). In contrast, shPLOD2-BGC823 cells increased the sensitivity to 5-FU with a significant decrease of IC50 when compared to that of the NC and shControl BGC823 cells (Figure 2B). Moreover, to further confirm that PLOD2 could enhance 5-FU resistance in GC cells, Transwell assays were performed to assess the changes in migration and invasion of the cells that were treated with or without 5-FU (Figure 2C and D). As shown in Figure 2E and F, the percentage of migrated cells in OE-PLOD2-MGC803 cells was significantly higher than that in the NC and OE-Control MGC803 cell groups, while the migration was markedly inhibited in shPLOD2-BGC823 cells compared to NC and shControl BGC823 cells with or without 5-FU treatment. ATP-binding cassette (ABC) transport proteins, such as P-glycoprotein (P-gp), multidrug resistance-associated protein 1 (MRP1) and breast cancer resistance protein (BCRP), can transport chemotherapy drugs, including 5-FU, out of tumor cells to decrease the cytotoxicity of drug 18,19. We examined the expression levels of P-gp, MRP1, and BCRP by Western blot analyses. Only BCRP expression was higher or lower in OE-PLOD2-MGC803 cells or shPLOD2-BGC823 cells, respectively, than in the NC and control cell groups when treated with 5-FU. P-gp and MRP1 expression showed no significant changes (Figure 2G and H). Taken together, these findings provide evidence that PLOD2 could decrease drug sensitivity to enhance 5-FU resistance in GC cells by regulating BCRP.

### PLOD2 inhibits 5-FU-induced apoptosis in GC cells

To further determine the effect of PLOD2 on 5-FU-induced apoptosis, flow cytometry analysis was performed in MGC803 and BGC823 cells with or without 5-FU. After 1 μM 5-FU stimulation, apoptosis was significantly decreased in the PLOD2-overexpressing MGC803 cells, and apoptosis was significantly promoted in the PLOD2 knockdown BGC823 cells; the apoptosis rate did not significantly change without 5-FU. We measured the protein levels of apoptosis-associated genes, including pro-apoptotic (Bax) and anti-apoptotic genes (Bcl2) by Western blot. As expected, after 5-FU treatment, upregulated expression of Bax but significantly downregulated expression of Bcl2 was observed in shPLOD2-BGC823 cells compared with the NC and control cells; the opposite was observed in OE-PLOD2-MGC803 cells. The expression of apoptosis-related proteins did not differ significantly without 5-FU. Altogether, these results demonstrated that PLOD2 inhibited cell apoptosis under 5-FU treatment by affecting the expression of Bax and Bcl2.

### Knockdown of PLOD2 increases 5-FU sensitivity in tumors *in vivo*

**Figure 4:**
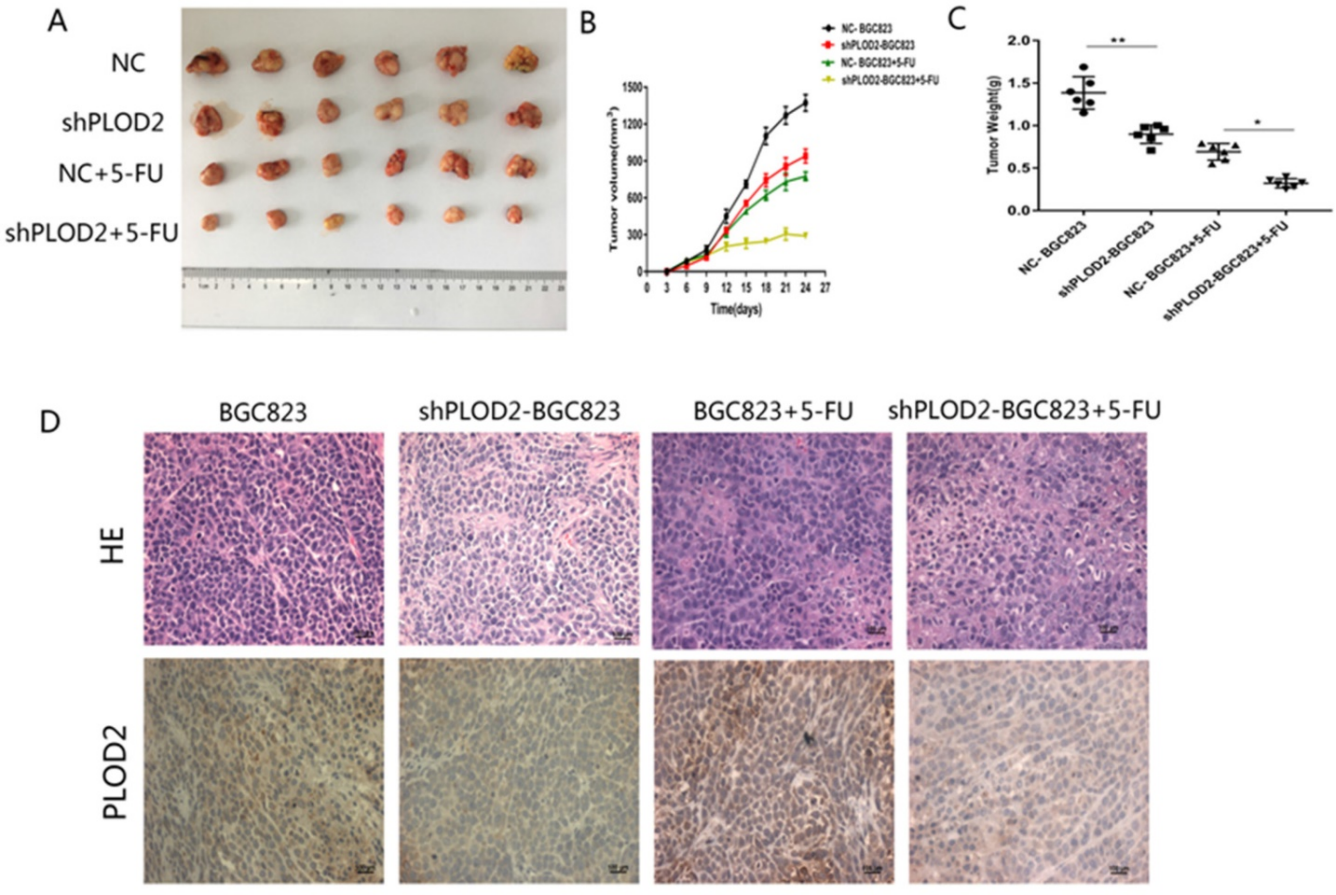
** Knockdown of PLOD2 increases 5-FU sensitivity in tumors *in vivo*. (A)** Xenograft tumors were stripped from nude mice and imaged. **(B)** The tumor volume in nude mice of NC and shPLOD2 BGC823 groups injected PBS or 5-FU. **(C)** The tumor weight in nude mice of NC and shPLOD2 BGC823 groups injected PBS or 5-FU. **(D)** Representatives of hematoxylin staining PLOD2 staining in tumor tissue derived from injected mice (Magnification: ×400). Data were presented as the means ± SD from six tumor samples. *P < 0.05, **p < 0.01.

To further confirm the functional role of PLOD2 in 5-FU resistance *in vivo*, NC-BGC823 and shPLOD2-BGC823 cells were subcutaneously injected into nude mice to generate a xenograft model, and then the mice were treated with 5-FU or PBS. The data revealed that the tumor growth of the shPLOD2-BGC823 group was dramatically slower than that of the NC-BGC823 group after 5-FU or PBS treatment (Figure 4A), as evidenced by the reduction in tumor weight (Figure 4B) and tumor volume (Figure 4C). We further detected PLOD2 expression in tumors by immunohistochemical (IHC) analysis. As shown in Figure 4D, the level of PLOD2 expression in tumors from the shPLOD2-BGC823 group was much lower than those from the NC‐BGC823 group treated with or without 5-FU. Therefore, our results indicate that downregulation of PLOD2 facilitated the sensitivity of GC cells to 5-FU *in vivo*.

## Discussion

Presently, 5-FU is still an important chemotherapeutic drug applied for the treatment of GC 20. However, the development of drug resistance leads to limited therapeutic effectiveness for GC patients in the clinic. Elucidating the molecular mechanism underlying 5-FU resistance could contribute to developing reasonable and effective therapies to overcome 5-FU resistance 21,22. PLOD2 is a collagen-modifying enzyme. Collagen is a protein that provides scaffolds in ECM 23. The ECM is produced by multiple TME cell types and weaves an intricate fiber network not only providing structural support but also regulating the physical and biochemical properties of the tumor microenvironment, thereby regulating the polarity, migration and signaling of cancer cells 24. The tumor microenvironment not only plays a key role in tumor progression but also is associated with chemoresistance 25,26. In breast cancer, attachment of malignant cells to the ECM alters their polarization and causes resistance to etoposide-induced apoptosis 27. Cancer-associated fibroblasts (CAFs) are the most populous cell type within the tumor microenvironment of many solid and hematological malignancies. CAFs from bortezomib-resistant patients are resistant to the drug *in vitro* and prevent bortezomib-induced apoptosis of cocultured multiple myeloma (MM) cells 28. Tumor-associated fibroblasts (TAFs) are another kind of cell type within the tumor microenvironment. Data have shown that TAFs play significant roles in the therapeutic sensitivity of tumors and that therapeutic targeting of TAFs results in increased chemotherapeutic sensitivity in colorectal cancer 29.

PLOD2 is overexpressed in multiple cancers, including GC 17. However, no study has shown its role in GC chemotherapy. In this study, we investigated the effect of both high and low PLOD2 expression on chemoresistance to 5-FU. We found that PLOD2 overexpression decreased the sensitivity of MGC803 GC cells to 5-FU, while knockdown of PLOD2 significantly enhanced the sensitivity of BGC823 GC cells to 5-FU. The knockdown of PLOD2 in BGC823 significantly decreased the IC50 values of 5-FU. It also contributed to reducing the cell migration and invasion of GC cells with or without 5-FU treatment. Interestingly, when treated with 5-FU, the knockdown of PLOD2 promoted apoptosis, but there was no significant change in the absence of 5-FU. The opposite results appeared in PLOD2-overexpressing MGC803 GC cells. In addition to *in vitro* experiments, we performed *in vivo* verification experiment using BGC823 and shPLOD2-BGC823 cells and found that the knockdown of PLOD2 increased the growth inhibition in response to 5-FU in xenografted tumors from in the nude mouse model. Therefore, PLOD2 was confirmed to play a critical role in 5-FU resistance.

The molecular mechanisms of drug resistance are complex and involve drug metabolism, drug target alteration, drug efflux, DNA damage repair, and anti-apoptosis inhibition 30. One of the most studied mechanisms of cancer resistance involves reducing drug accumulation by enhancing efflux. ABC transporter family members, including P-gp, MRP1 and BCRP, can achieve this efflux 31. To gain further insight into the molecular mechanisms of PLOD2-mediated 5-FU resistance, we evaluated the P-gp, MRP1 and BCRP protein levels in each group. The results demonstrated that P-gp and MRP1 had no significant changes, but the expression of BCRP was downregulated in PLOD2 knockdown BGC823 cells and upregulated in PLOD2-overexpressing MGC803 cells treated with 5-FU. 5-FU is known as a substrate for BCRP. In previous studies, downregulation of GLI2 sensitized cancer cells to 5-FU treatment, and GLI2 mediates cancer cell resistance to 5-FU through direct regulation of BCRP 32. Overexpression of the multidrug resistance transporter BCRP *in vitro* has been shown to cause resistance to 5-FU, which is a component of the most commonly adopted regimens for treating colorectal cancer 33. Our mechanistic studies revealed that PLOD2 appears to be resistant to the therapeutic characteristics of 5-FU in GC cells via upregulation of BCRP. In addition, we analyzed the apoptosis-related protein levels and found that the level of Bax protein is significantly increased and Bcl2 protein was significantly decreased after treatment of cells with 5-FU when PLOD2 was knocked down, and the opposite conclusion was obtained when overexpressing PLOD2. Therefore, we demonstrate that PLOD2 confers resistance to 5-FU-induced apoptosis in GC cells by affecting the expression of Bax and Bcl2. However, further in-depth studies are needed to elucidate other molecular mechanisms of 5-FU chemoresistance that are induced by PLOD2 overexpression.

Overexpression of PLOD2 upregulated the expression of BCRP protein and inhibited apoptosis by decreasing BAX and increasing the level of Bcl2, enhancing the resistance of gastric cancer cells to 5-FU. Knockdown of PLOD2 downregulated the expression of BCRP protein, while increasing BAX and decreasing the level of BCL2 promoted apoptosis, reducing the resistance of gastric cancer to 5-FU. Collectively, these data revealed that PLOD2 contributed to increasing resistance to 5-FU in GC cells by upregulating BCRP and decreasing 5-FU-induced apoptosis. These results provide new insights into the roles of PLOD2 as a promising therapeutic target for 5-FU resistance in GC.

## Figures and Tables

**Figure 3:**
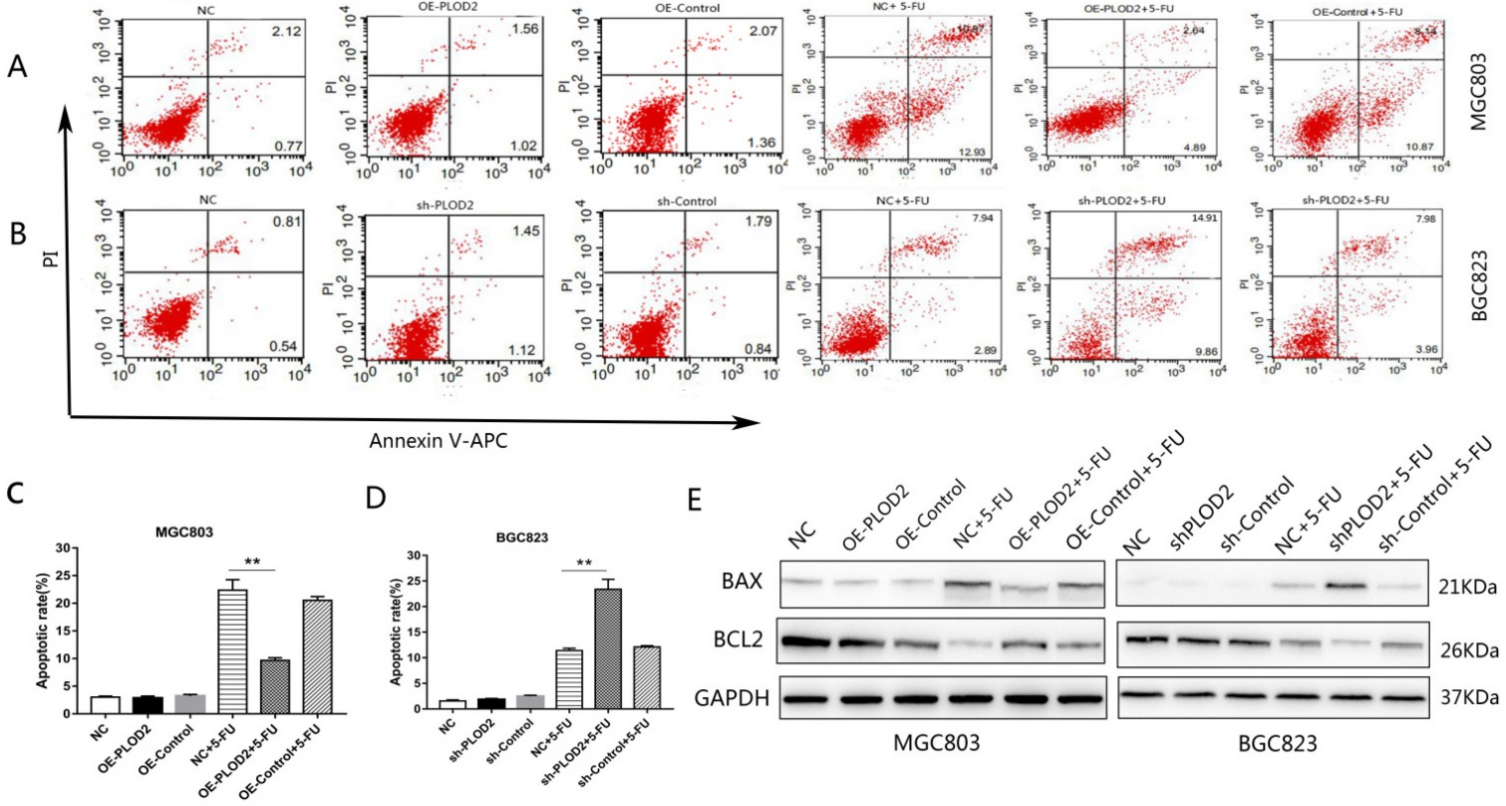
** PLOD2 inhibits 5-Fu-induced apoptosis in GC cells (A,B)** Flow cytometry analysis was performed in MGC803 and BGC823 cells with or without 5-FU. **(C,D)** The cell apoptosis rate in each group. **(E)** Protein bands of Bax and Bcl2 in MGC803 and BGC823 cells measured by Western blot analysis. The results represent the mean ± SD of 3 independent experiments. *P < 0.05, **p < 0.01.
